# Synthesis of Carbon Nanotubes (CNTs) from Poultry Litter for Removal of Chromium (Cr (VI)) from Wastewater

**DOI:** 10.3390/ma14185195

**Published:** 2021-09-10

**Authors:** Noor Haleem, Yousuf Jamal, Shahid Nawaz Khan, Muhammad Anwar Baig, Maryam Wahab, Xufei Yang

**Affiliations:** 1 Institute of Environmental Sciences and Engineering (IESE), National University of Sciences and Technology (NUST), Islamabad 44000, Pakistan; yousufjamal.icet@pu.edu.pk (Y.J.); ma_baig@lycose.com (M.A.B.); 2 Agriculture and Biosystems Engineering Department, South Dakota State University, Brookings, SD 57007, USA; xufei.yang@sdstate.edu; 3 Institute of Chemical Engineering & Technology (ICET), University of the Punjab, Lahore 54590, Pakistan; 4 Institute of Geographical Information Systems (IGIS), National University of Sciences and Technology (NUST), Islamabad 44000, Pakistan; snawaz@igis.nust.edu.pk; 5 Atta Ur Rahman School of Applied Biosciences (ASAB), National University of Sciences and Technology, Islamabad 44000, Pakistan; Mwahab.mshcb06asab@nust.edu.pk

**Keywords:** poultry litter, carbon nanotubes, catalyst, experimental conditions, adsorption

## Abstract

Pakistan, an agricultural country, raises 146.5 million commercial and domestic poultry birds, which generate around 544,831 tons of waste per year. This waste finds its final disposal in agricultural land as soil fertilizer or disposal site amendment. The usage of poultry litter for this purpose is uncontrolled, which results in environmental degradation such as emission of greenhouse gases, e.g., methane. However, alternative options such as thermochemical conversion of poultry litter can offer better solutions where this waste can be used as a low-cost carbon source for the synthesis of Multiwalled Carbon Nanotubes (MWCNTs). In this study, efforts were made to utilize this cheap and plentiful carbon source for the synthesis of CNTs in the presence of Ni/Mo/MgO as a catalyst, through pyrolysis. For a better yield of carbon product, the optimum ratio for the catalysts (Ni/Mo/MgO) was found to be 4:0.2:1. Furthermore, the process parameters were also optimized for better carbon yield. A good yield of CNTs resulted from a pyrolysis time of 12 min, a temperature of 825 °C, and a catalyst weight of 100 mg. The structure and morphology of the produced nanotubes were confirmed through X-ray Diffractometer (X-RD) and Scanning Electron Microscopy (SEM). The environmental application of the nanotubes was tested in a synthetic chromium solution in the lab using a batch experiment. Different experimental conditions (pH, adsorbent dosage, and contact time) were optimized to improve the adsorption of Cr (VI) by carbon nanotubes and a UV-Visible spectrophotometer was used at 540 nm to measure the absorbance of Cr (VI). The results showed that up to 81.83% of Cr (VI) removal was achieved by using 8 mg of CNTs at pH 3 with 400 rpm at 180 min of contact time. Thus, it was concluded that poultry litter can be a useful source for the synthesis of MWCNTs and thereby removal of Cr (VI) from industrial tanneries’ wastewater.

## 1. Introduction

In Pakistan, the poultry sector is one of the vibrant segments of the livestock division. According to 2016–2017 statistics, 85.86 million domestic poultry and 60.6 million commercial poultry were produced [1]. In Pakistan, the poultry industry has a vital status in the rural economy considered the second largest industry and an income source for millions of people. [2]. Asia is the top regional exporter of prepared chicken products, delivering more than 800,000 tons in 2014, while Europe is the main buyer, purchasing more than one million tons in that year [3]. Organic waste from poultry is mainly the litter, which consists of several elements such as chicken urine and feces. However, there is also a mixture of several items such feed sawdust and bedding materials such as wood shavings in poultry litter. The quality and composition of litter varies with different elements such as diet, dietary supplements, storage of litter, and management practices of the poultry farm [4]. In the literature, quantified data about poultry litter produced in a 42 day cycle fluctuate between 1.5 and 5.7 kg per bird [5]. The average poultry litter production is 3.72 kg/bird. Common poultry litter for chicken is normally used several times, which helps to reduce the volume of generating beds [3]. Poultry litter generated by a single broiler unit usually varies due to several parameters such as feed intake by chickens, management scenarios (bedding material), and feed digestibility [6].

For centuries, poultry litter has been used as a fertilizer due to its organic properties for plant growth; it is rich with several nutrients which are required for plants [7]. Macronutrients such as nitrogen, phosphorus, and potassium (NPK) and micronutrients that are required in little amounts for crop growth such as copper, zinc, etc., are present in litter produced by poultry [8]. Poultry litter has been also used as a mulching material, since poultry litter has properties of conservation of soil moisture. During summer when it is hot, it also protects the surface, which feeds the roots [9]. It is also used as a low-cost renewable resource for producing activated carbon by burning the litter to at least 700 °C resulting in the formation of a lattice-like carbon particle structure. Activated carbon is used for the adsorption of contaminants in wastewater [10,11]. Along with this, several physicochemical and hydroponic techniques have been reported for metals removal [12,13,14].

Transition metals reinforced on silica, mesoporous silica, calcium carbonate, zeolites, or magnesium oxide are used as catalysts for the growth of MWCNTs [15]. Many catalysts have been studied to improve and change the structure and properties of MWCNTs to increase their yield. Various catalysts, i.e., Ni, Co, Mo, and Fe have been used for the growth of MWCNTs. Supported metal catalysts have been formed by using the impregnation method. Keeping in view all parameters related to the chemistry of metal is important while performing the impregnation method for manufacturing a supported metal catalyst [16].

Nanomaterials are progressively used for diverse modern technologies and CNTs are among the most prominent. MWCNTs are made of graphene sheets of a hexagonal structure rolled up into a nanoscale tube. They vary in their lengths up to a million times as compared to their diameter, which is up to 0.4 nm [17].

MWCNT synthesis and its utilization have been studied across the world in the last few years with extraordinary attention. Today, MWCNTs and their use in different areas are discussed widely in scientific circles. Usage of MWCNTs for removal of different wastes such as organic waste, inorganic waste, and radionuclides from wastewater is very common. Several researchers reported the extensive applications of these materials in a variety of areas such as science, engineering, energy storage, electrochemical super capacitors, etc. MWCNTs have exceptional properties in terms of physical, electrical, and mechanical aspects and thus have encouraged new technologies. Other factors that make them different from other materials are their density (nearly half the density of aluminum), stiffness, and large surface area [18].

The hydrocarbon sources used for the growth of MWCNTs include mainly ethylene, methane, and acetylene. Liquid hydrocarbons such as benzene, xylene, cyclohexane, and alcohol are also being used as CNTs precursors. Carbon nanotubes can also be generated from solid biomass waste such as rice straw [19] and propylene bottles [20].

Research on the direct growth of MWCNTs has been reported using in situ techniques in which a catalyst, i.e., transition metal alloy is taken and a carbon containing material is passed through it. However, the only challenge is that catalyst creation is very lengthy, tiresome, and too costly, which may increase the cost of the MWCNTs. Recently, magnetic separation techniques have increasingly come to the attention of scientists [21]. The applications can be seen in many fields such as medicine, cell biology, analytical chemistry, and environmental engineering. The synthesized magnetic MWCNTs can be well dispersed in water. Moreover, the removal of MWCNTs, from a medium with the help of a magnet is convenient [22]. Removal of heavy metals, organic, and inorganic contaminants using biochar [23] and magnetic CNTs [24] as adsorbents has been reported.

There are two objectives for this study. First, poultry litter as a carbon source is used to produce MWCNTs with a Ni/Mo/MgO catalyst. Response Surface Methodology (RSM) was used to optimize the parameters (such as temperature, time, and weight of the catalyst) both for catalyst preparation and growth of MWCNTs. The second objective of this research was to analyze the adsorption behavior of CNTs for Cr (VI) adsorption. Effects of factors, such as system pH, adsorbent dosage, and adsorbent contact time were investigated. pH effects on the removal of Cr (VI) were also investigated.

## 2. Materials and Method

Commercial poultry units in Islamabad, Pakistan were reached for the collection of poultry litter. Fresh litter was collected since the flock was just removed from the poultry farms. Litter was then transported to the Institute of Environmental Sciences and Engineering (IESE). The bedding material was constituted of sawdust, no medicine was given to the birds during the growth phase. The initial poultry litter sample of 3 kg was kept in closed bags. For the synthesis of the catalyst, Nickel Nitrate hexahydrate (Ni(NO_3_)_2_·6H_2_O), Ammonium Molybdate tetrahydrate (NH_4_)_6_Mo_7_O_24_·4H_2_O), Magnesium nitrate hexahydrate (Mg(NO_3_)_2_·6H_2_O), Citric acid, Hydrochloric Acid (HCl), Sodium Hydroxide (NaOH), and Ethanol were purchased from Sigma Aldrich, St. Louis, MO, USA. The chemicals used in this study were of analytical grade and were used in the form they were received. In all the experimental runs, distilled water was used.

### 2.1. Synthesis of Catalyst

The wet impregnation method was used for the synthesis of catalysts. The solution of 116.28 g of Ni (NO_3_) _2_·6H_2_O, 24.71 g of (NH_4_)_6_ Mo_7_O_24_·4H_2_O, and 25.64 g of Mg (NO_3_)_2_ 6H2O was added to 200 mL of distilled water and stirred for 1 h on a magnetic hot plate. The mixture was heated to 90 °C up to 1 h after the addition of two grams of anhydrous citric acid. The resultant mixture was left on a hot plate for evaporation. The viscous slurry was oven-dried at 120 °C for 12 h. The material was ground into fine powder form and calcinated in the furnace of a tube-like structure at 600 °C for 2 h (10° rise/min) [25].

### 2.2. Synthesis of CNTs

The raw material used for the synthesis of CNTs was poultry litter. The litter was dried in the oven at 120 °C for 3 h and ground. Four grams of poultry litter was then mixed manually with 2–8 mg of the catalyst. The mixture was then placed in a porcelain boat (volume 80 mL) and combusted using an electrical furnace at 700–950 °C in a continuous flow of helium gas to provide an inert atmosphere. The porcelain boats were removed after 12 min from the tube furnace. During the decomposition of organic compounds, carbon molecules are deposited on the nickel catalysts placed on magnesium oxide as supporting material. In the reported literature, the synthesis is carried out within the temperature range of 750–850 °C [26].

### 2.3. Process Parameter Optimization with Response Surface Methodology

The Response Surface Methodology (RSM) with Box–Behnken design was used to maximize the yield of CNTs by optimization of process parameters. In contrast to conventional methods, the interaction between the processes’ variables was determined by statistical analysis of the response surface methodology. To optimize the MWCNTs’ synthesis, a two level half factorial design with three central points was preferred. The parameters optimized for this study were (a) Reaction Time, (b) Temperature, and (c) Catalyst concentration. For synthesis of the CNT samples, 15 experimental runs were automatically defined by the statistical software. For finding the optimum conditions for the reaction, the response from each experimental run was statistically analyzed based on yield [27].

### 2.4. Purification of MWCNTs

Impurities such as residual catalyst and amorphous carbon must be removed from CNTs. This requires chemical and thermal treatment. Catalyst particles were removed by an ultrasonication process in which 37% concentrated hydrochloric acid was used and stirred for 2 h on a magnetic hot plate. The resultant mixture was filtered by vacuum after dilution with deionized water. Distilled water was used to neutralize the pH of the solid carbon product. Oxidation of MWCNTs was performed in a tube furnace at a temperature of 400 °C for a duration of 2 h, following the method explained by Vivekchand et al. [28].

### 2.5. Functionalization of CNT

A solution of 200 mL of 6.0 M HNO_3_ (70%) was prepared and 0.7 g MWCNT was spread in this solution by the dispersion method. An ultrasound bath was used for 20 min to maintain the dispersion process. The dispersion was magnetically stirred for 12 h. This period was under nitric acid reflux and under different temperatures ranges of 50, 70, 90, and 110 °C. The dispersion was cooled down at room temperature after removing it from the hot plate after 12 h of treatment. Centrifugation of the dispersion was performed for 10 min at 4000 rpm. This process separated the supernatant from the mixture by sediment solid residues. The supernatant was filtered under vacuum by using a 0.2 μm acetate membrane filter. Distilled water was used to wash the solid residue to remove extra nitric acid from the sample. This washing process continued until the pH of the filtrate became neutral. Finally, the MWCNTs were dried for further analysis [29].

### 2.6. Adsorption of Cr (VI)

In 100 mL distilled water, 0.2829 g of potassium dichromate (K2Cr2O7) was dissolved, and the Cr (VI) stock solution of 1000 mg L^−1^ was produced. To prepare various solutions of different concentrations of Cr (VI), the stock solution was diluted with distilled water. Adsorption analyses were conducted by blending 2, 4, 6, and 8 mg of MWCNTs with 50 mL Cr (VI) solutions of 100 ppm in a 100 mL volumetric flask. The solutions were agitated at 500 rpm over various contact times from 30 min to 210 min with an increment of 30 min. Cr (VI) solution pH values were tweaked to 2.0–7.0 by using 1.0 M HCl and 1.0 M NaOH solution [30].

The effect of the adsorbent dosage was determined, and a pH value of 3 and the initial Cr (VI) concentrations in the solutions were used. An acetate membrane filter of 0.2 μm thickness was selected to separate the aqueous phase. A UV-Visible spectrophotometer was used to detect the absorbance of Cr (VI).

### 2.7. Characterizations

#### 2.7.1. X-ray Diffraction

To obtain the pattern of X-ray diffraction of the catalyst, an X-ray diffractometer (Theta/Theta STOE Jeol, Freising, Germany) was used. The samples were prepared by pressing the powders between two glass slides into a flattened sheet. The radiation source CuK was used for taking X-ray patterns and 40 kV and 40 mA was supplied to the X-ray generator. The patterns were recorded at 2θ from 20° to 70°.

#### 2.7.2. Scanning Electron Microscopy (SEM)

SEM (Jeol JSM-6490A, Tokyo, Japan Analytical scanning electron microscope) was used in this study to analyze the surface morphologies of the samples. The samples were coated with a thin layer of the conducted material (gold). They were then imaged at ×20,000, ×35,000, and 75,000 magnifications, the accelerating voltage was 10–15 kV. A focused high beam of electrons interacted with the surface of the sample and generated secondary electron, backscattered electron, and characteristic X-ray signals. The detector processed the signals and displayed the images on a screen [31].

#### 2.7.3. Transmission Electron Microscopy (TEM)

The sample was characterized using a Jeol JEM-100cx transmission electron microscope. An accelerating voltage of 20 kV was used for characterization. The prepared sample was first sonicated in ethanol for 5 min at room temperature, and then a drop of dispersion was deposited on a Cu/Rh grid covered with a vinyl polymer (formvar), and the grid was dried overnight under vacuum [32].

#### 2.7.4. Raman Spectroscopy

Raman spectroscopy of the synthesized CNTs was performed using a Raman Spectrophotometer uRaman-532 TEC-Ci Technospex, Bukit Merah, Singapore, in ambient conditions. The spectrum was recorded using an incident laser with an excitation wavelength of 532 nm, and the samples were exposed for 60 s.

## 3. Results and Discussion

### 3.1. Initial Analysis of Poultry Litter

#### 3.1.1. Moisture Content

The moisture content (*w*/*w*%) of the poultry litter (PL) was determined as the weight loss of about 91.87 g of the sample when dried in an oven at 105 °C for 8 h. The total moisture content of raw material was 54.06% as illustrated in Table 1.

(1) $$M=\frac{W2-W1}{W2}\times 100$$

Average of the above dry weight = 91.87. Putting this into Equation (1)

(2) $$M=\frac{200-91.87}{200}\times 100=54.06\%$$

#### 3.1.2. Elemental Analysis

**Figure 1:**
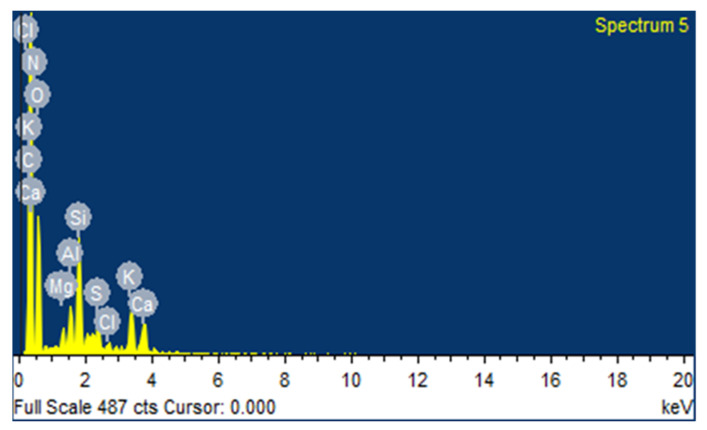
EDS of poultry litter through SEM.

The spectroscopic method (Section 3.7.3) was used to determine the elemental concentrations in the poultry litter as depicted in Figure 1. Analysis of the surface composition of the poultry litter is illustrated in Table 2. Concentrations of C, N, P, O, CI, Na, Ca, Mg, S, and Si were found in PL samples without bedding material (not mixed with bedding material).

### 3.2. Optimization of Mole Ratio of Catalyst Precursors

To characterize the effects of the Ni/Mo mole ratio on the carbon yield RSM was applied. The outcome of each independent variable (Ni/Mo/MgO mole ratio) on the dependent variable (carbon yield) was subjected to linear regression. Multiple linear regression was used to explain the association between one continuous dependent variable (carbon yield %) and three independent variables (concentration of Ni, Mo, and MgO). To confirm the effects of the catalyst on the carbon yield, various concentrations of Ni, Mo, and MgO were studied as shown in Table 3. For optimization of a process parameter, 15 trial runs were executed. The data in Table 3 show that Mo, MgO, and Ni contents varied from 0.2 to 0.6, 1.0 to 3.0, and 2.0 to 6.0 moles, respectively. With the increase in Ni concentration up to 4 moles, the carbon yield experienced a sharp increase. Further increase in Ni did not lead to further increase in carbon yield; rather, it tended to decrease with a further increase in Ni. This sudden decrease indicated that Mo and Ni should be in a suitable ratio for optimized carbon yield. It was also observed that for an increased yield of carbon more moles of Ni are required than Mo. Secondly, CNT growth has not been observed on Mo and was only detected on Ni metal ions, which means Mo solely enhances Ni ions’ activity. This shows that for the carbon product formation only a small quantity of Mo is required. In this study, four moles of Ni in the catalyst (Ni4Mo0.2MgO1) provided the maximum yield of carbon. Variations in carbon yield could be attributed to the catalysts having the same Ni/Mo mole ratio and different MgO concentrations. This also illustrates another point: an increased quantity of MgO did not lead to an increase in carbon yield. This is due to the fact that MgO only provides support for Mo and Ni ions.

Table 4 explains the variations in carbon yield due to the change in the Ni/Mo mole ratio because of the analysis of variance (ANOVA). It was observed that the suitable model to analyze the response of carbon yield was the quadratic model. From the *p*-value, F-value, and coefficient of determination (R^2^) of the model, the suitability of the model was established. The importance of the coefficient is determined by the value of *p,* which means the smaller the *p*-value the more important the coefficient is. In this model, an F-value of 117.97 and *p* < 0.0001 confirmed the significance of the model. R^2^ = 0.9953 suggested the fitness of the quadratic model [27,33].

Carbon Yield = −3.60000 + 23.45066Ni + 0.328947Mo − 7.59868Mg − 0.947368Ni * Mo − 0.288158Ni * Mg − 4.39474 Mo * Mg − 2.42368 Ni² + 0.065789 Mo² + 2.30526 Mg²(3)

**Figure 2:**
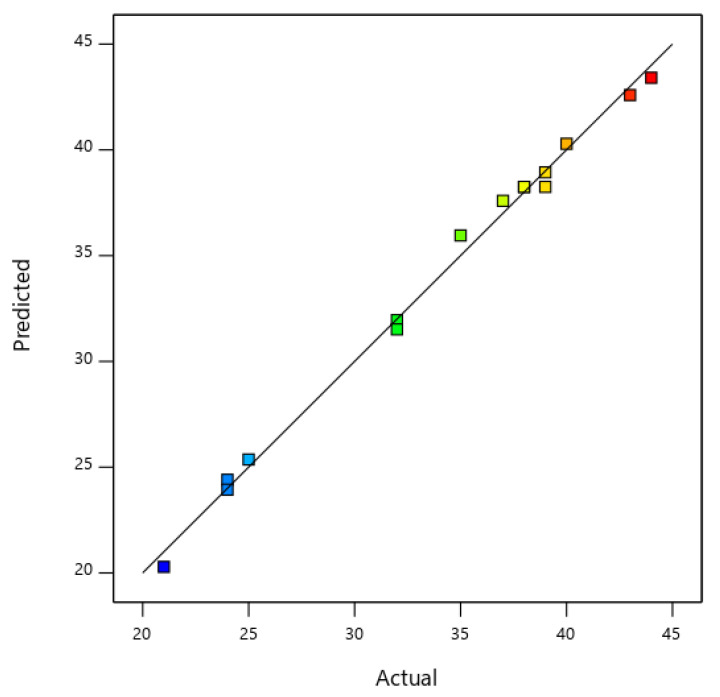
Carbon product predicted and actual yield derived from the RSM model.

**Figure 3:**
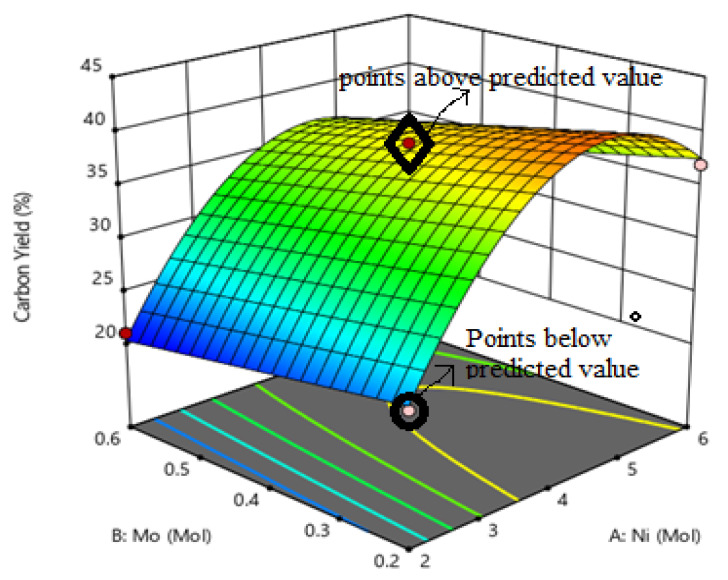
Plot showing catalytic activity of Ni, Mo, and MgO over the carbon yield.

Equation (1) with regard to actual factors was utilized to predict the response for different levels of each factor as shown in Figure 2. Figure 3 illustrates the 3D response of the catalytic activity of Ni, Mo, and MgO on the yield of carbon product.

### 3.3. Statistical Analysis and Modeling for CNTs Growth

For process parameters, the detection of the initial level is important to carry out. Therefore, optimization was performed using trial experiments. To optimize the process parameters, a full factorial design method and several runs were required. In this research, we executed various experimental runs and their analysis based on results described by [34]. In all the experimental runs, it was observed that less than 12 min of pyrolysis time led to the partial combustion of poultry waste while more than 20 min led to the fast oxidation of CNTs as shown in Table 5. CNT growth was not seen below 700 °C while quick combustion of raw material along with CNT oxidation was noted at above 900 °C. The lowest and highest values for poultry waste and catalyst weight were selected based on the porcelain boat’s volume employed in the experiments carried out in this study.

**Figure 4:**
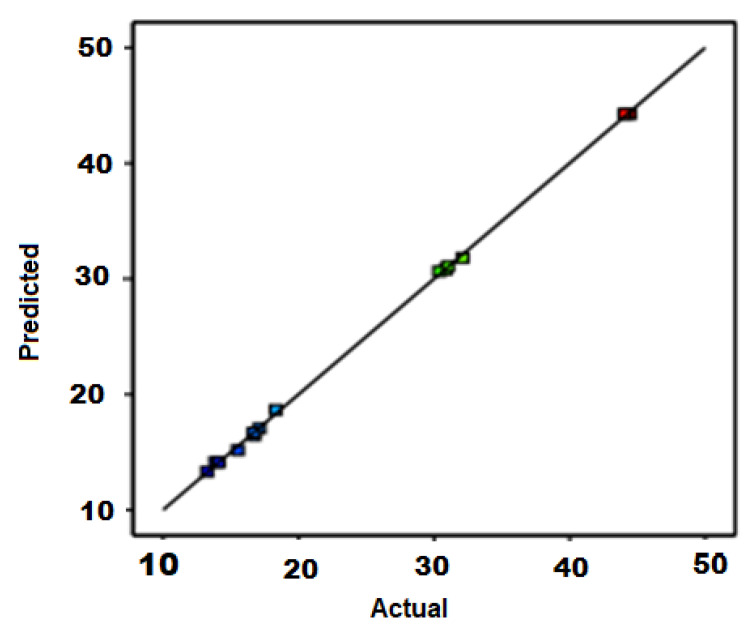
Actual and predicted carbon yield derived from the RSM Model.

Response optimization is influenced by various parameters, a response surface methodology (RSM) was used. Three different levels with the three variable experimental design of Box–Behnken were chosen. Fifteen experimental runs were performed to analyze the influence of the different variables on carbon yield. The correlation between the experimental and predicted yield of carbon product over different variables is shown in Figure 4. Reaction temperature (700–950 °C), poultry litter weight (2–4 g), catalyst weight (80–120 mg), and reaction time (5–20 min) were chosen to optimize the maximum yield of the carbon product. The optimized values for the synthesis of catalyst (Ni/Mo/MgO) with moles of Ni, Mo, and MgO for the growth of CNTs were 4:0.2:1. Such a result is consistent with Run 11 in Table 3, with the highest yield ratio of 44%. In the synthesis process of CNTs, carbon diffusion occurs with Ni and Mo nanoparticles. Moreover, Mo converts to Mo carbide. As the intensity of carbon diffusion on Ni and Mo nanoparticles increases, the carbon precipitation occurs on the Ni–Mo crystal plane and carbon nanotube bundles are produced.

**Figure 5:**
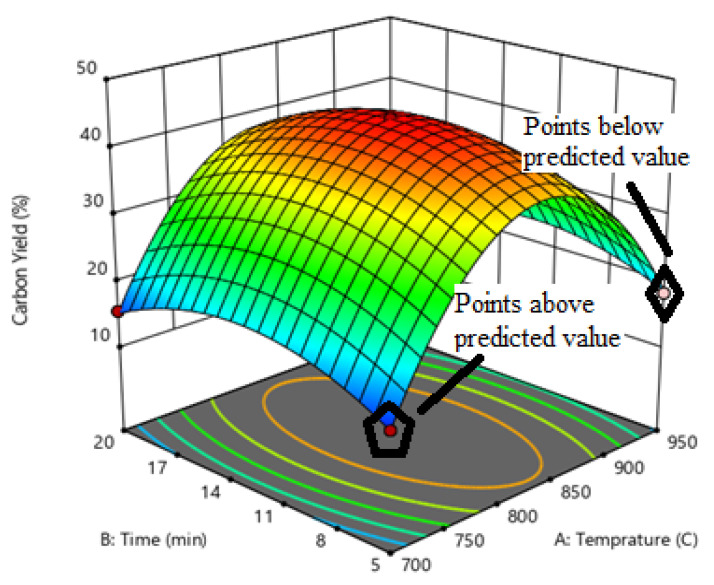
Effects of variables on carbon yield.

Three-dimensional responses showing the effect of variables on the carbon yield are shown in Figure 5. Table 6 depicts the effects of independent variables (various process parameters) on the dependent variable (carbon yield). The regression equation for the same is illustrated in Equation (4).

Carbon Yield = −1129.58015 + 2.34585 Temperature + 3.45479 Time + 3.57753 Catalyst Weight −0.000803 Temperature * Time − 0.000021 Temperature * Catalyst Weight − 0.000983 Time * Catalyst Weight −0.001407 Temperature² − 0.108970 Time² − 0.017649 Catalyst Weight²(4)

Equation (4), with regard to actual factors, can be utilized to predict the response of dependent variable for several given levels of each factor. Developed on model fitting and multiple linear regression, the appropriate model was selected to resolve the interactions of the response function with the selected factors.

(5) $$Y=\mathrm{ao}\sum_{i=1}^{4}\mathrm{aixi}+\sum_{i=1}^{4}\mathrm{aiix}2i+\sum_{i=1}^{4}\mathrm{aijxixj}\;$$

where Y = Response, a_0_ = Constant coefficient, a_i_, a_ii,_ and a_ij_ = are the coefficients predicted by regression for linear, quadratic, and crossproduct effects of X_1_, X_2_, and X_3_, respectively.

In this study, the variables X_1_, X_2_ and X_3_ were allocated for reaction temperature [A], reaction time [B], weight of catalyst [C], and time of reaction [35].

### 3.4. Temperature Effects on Growth of CNTs

**Figure 6:**
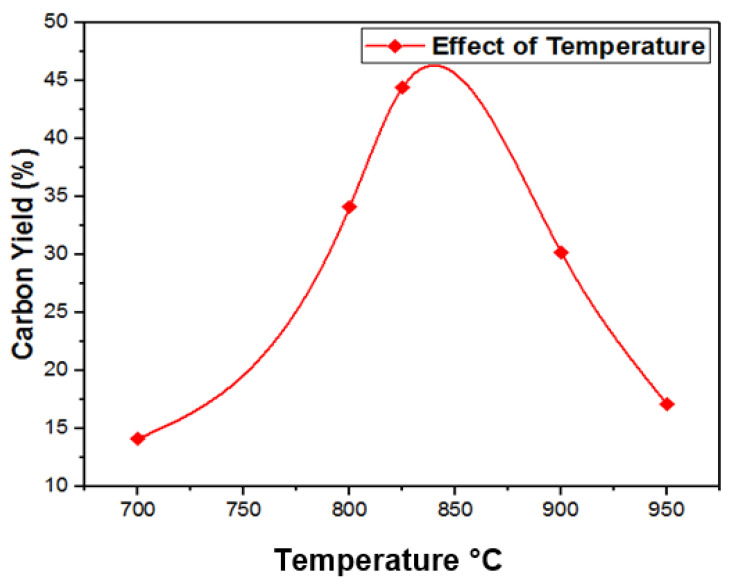
Changes in percent carbon yield with change in temperature.

To analyze the effects of temperature on carbon yield a three-dimensional response surface curve was used as illustrated in Figure 6. To analyze the potential effects of the independent variables on the dependent variable (carbon yield), curves were obtained. The carbon yield was maximized with the catalyst load of 100 mg at around 800–850 °C. However, at higher temperatures (850–900 °C), vapors of hydrocarbon passed out of the crucible quickly, which caused a reduction in the hydrocarbon source for CNT growth.

### 3.5. Reaction Time and CNTs Growth

**Figure 7:**
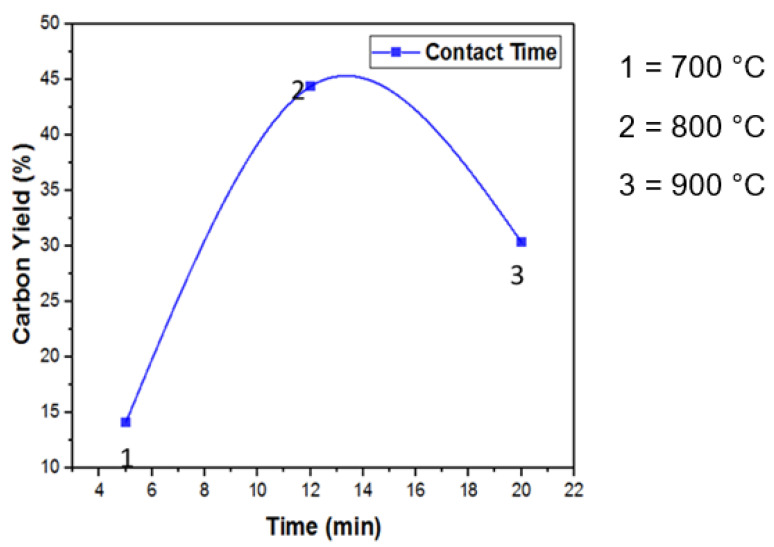
Effect of optimized contact time on carbon yield.

Figure 7 illustrates how the reaction time affects CNT growth. In all the experiments, the poultry waste weight (4 g) was kept constant. It was observed that CNT yield continuously decreased as the reaction time within the tube furnace increased. For 10–12 min, the contact of hydrocarbon vapors with catalysts particles was observed after which the carbon-rich vapors started leaving the porcelain boat. Twelve minutes of contact time was optimal. The exposure of samples to a higher temperature for a longer time resulted in the oxidation of hydrocarbons, which was the major cause of the reduction in CNT yield [36].

### 3.6. Catalyst Characterization

#### 3.6.1. X-ray Diffraction (XRD)

**Figure 8:**
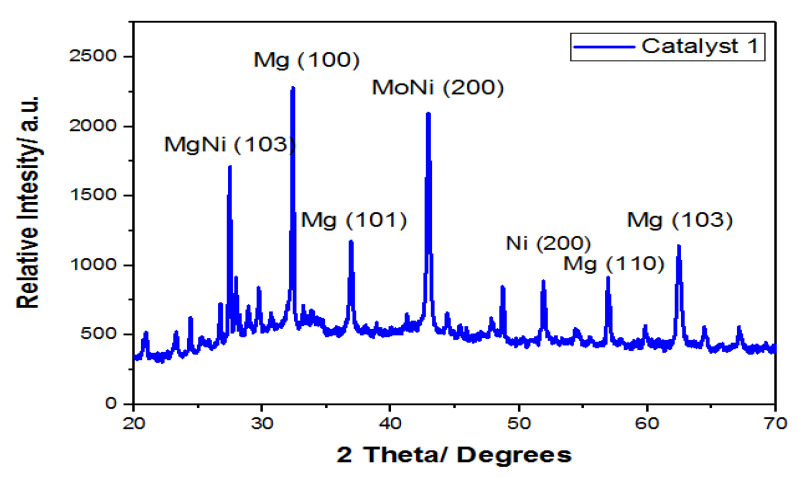
XRD of the optimized molar ratio of Ni/Mo/Mg (4:0.2:1) catalyst.

Figure 8 illustrates the Ni/Mo/MgO (4:0.2:1) catalyst diffraction. In these patterns, the intense peaks at 37.30° and 43.28° corresponded to Mg and MoNi respectively. It was confirmed that Ni and Mo particles were well supported over the MgO matrix and sintering was not observed due to the presence of sharp peaks [37].

#### 3.6.2. Morphological Analysis of the Catalyst

**Figure 9:**
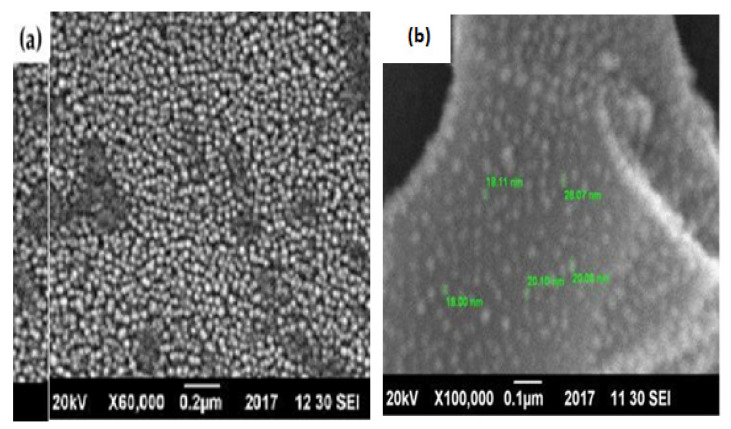
SEM of the catalyst with the ratio of Ni_4_Mo_0.2_MgO_1_. (**a**) Ni and Mo particles (**b**) Morphology of Ni/Mo/MgO catalyst particles with average size of 18 nm.

Scanning Electron Microscopy (SEM) procured from Jeol, Tokyo, Japan was utilized in this study to examine the surface morphologies of various samples. The morphology of the Ni/Mo/MgO catalyst particle with an average size of 18 nm at ×100, 000/- magnification is depicted in Figure 9b. The size of the nanoparticles did not depend on the specific magnification. A uniform catalyst layer with a good distribution of Ni, Mo, and MgO (4:0.2:1) particles was observed from the catalyst morphology. The metal particles appeared as individual crystals as well as in segregated form. Due to the dark and spherical shape of both Ni and Mo, it was challenging to discern the Ni and Mo particles as shown in Figure 9a. The Mg matrix well incorporated the Ni and Mo particles as shown from the microstructure of Ni/Mo/MgO. Several Mg–Mo and Ni–Mo phases for the Ni/Mo/MgO catalyst were shown by the XRD report; therefore, these results agreed with XRD result.

### 3.7. Characterization of CNTs

XRD, SEM, and HRTEM techniques were used to study the effects of the various mole ratios of Ni/Mo/Mg on CNT yield.

#### 3.7.1. X-ray Diffraction (XRD)

**Figure 10:**
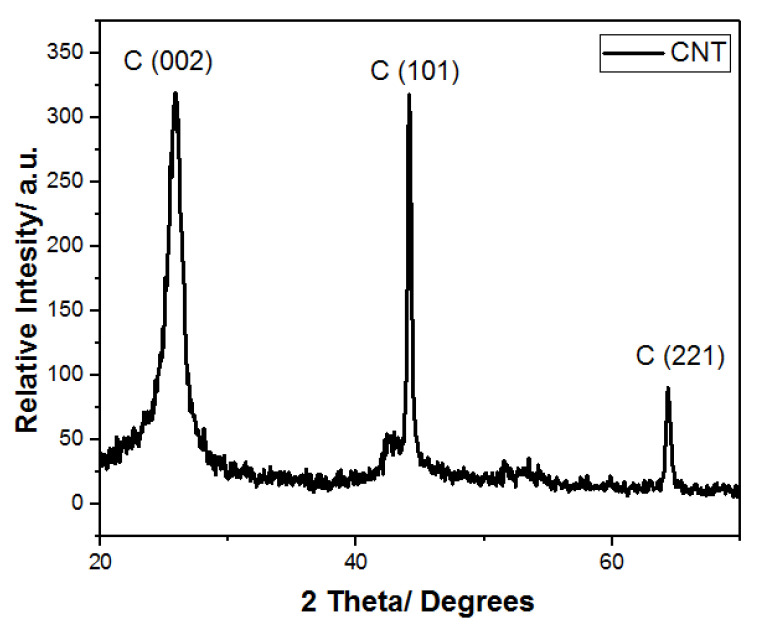
XRD of purified CNTs.

The XRD patterns of purified CNTs after synthesis over 0.1 g of Ni/Mo/MgO catalyst at 825 °C, time of combustion = 12 min, and 4 g of poultry litter are shown in Figure 10. The well-resolved graphite (0 0 2) peak at 2θ = 26.62° was observed, which indicates the growing of CNTs. During the synthesis of CNTs, carbon was diffused into Mo and No nanoparticles, and Mo was converted to a Mo carbide phase (MoC and Mo_2_C) [34]. Carbon nanotube bundles were formed due to the carbon atoms’ precipitation that occurred on the Ni–Mo crystal plane. Carbon atom precipitation on the surface of catalyst occurred when more carbon atoms diffused on Ni and Mo nanoparticles. For purified CNT, the XRD pattern as depicted in Figure 10 showed a sharp peak (0 0 2) with high intensity, which indicated the absence of amorphous carbon [38]. The peak at 2θ = 44.60° was caused by the presence of Ni particles in the CNT product. When the CNTs were purified, a decrease in peak intensity was observed. In the purification step, the partial removal of the catalyst particles through acid treatment were confirmed. However, it was challenging to ascertain the % removal of impurities from the characterization of purified CNT as it appeared similar to the CNTs, which were not purified [39].

#### 3.7.2. Raman Spectroscopy

**Figure 11:**
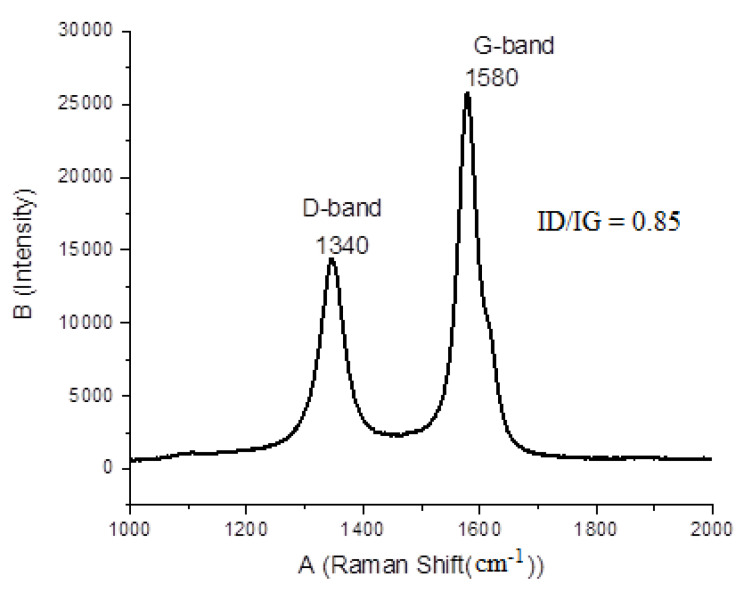
Raman spectra of CNTs synthesized at 825 °C with 12 min combustion time over a Ni4Mo0.2MgO1 catalyst.

Raman spectra results showed two characteristic bands at 1340 ± 5 cm^−1^ and 1580 ± 5 cm^−1^ as shown in Figure 11. The band at 1340 cm^−1^ was denoted as D (disorder) mode, which corresponded to the local structural impurities, defects, and disorder. The band at 1580 cm^−1^ was attributed to the G (graphite) mode, which corresponded to the degree of graphitization of the MWCNTs [40]. A minor displacement of D and G mode for the samples was observed and the peak intensity ratio was found to be 0.85. For purified CNTs, the lower peak intensity ratio was caused by graphene layer distortion. This occurred during treatment with concentrated HCl.

#### 3.7.3. Morphological Analysis of CNTs

**Figure 12:**
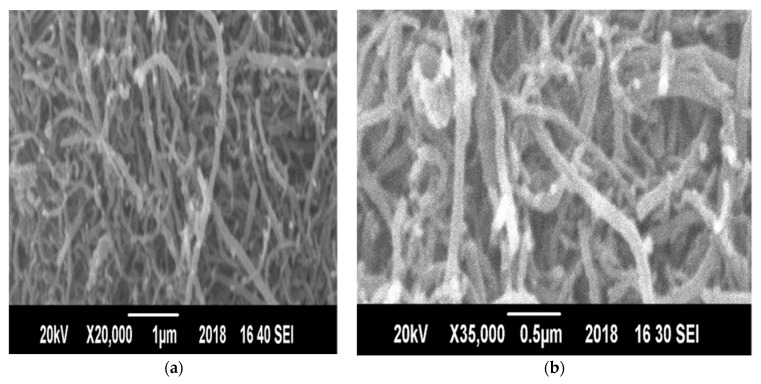

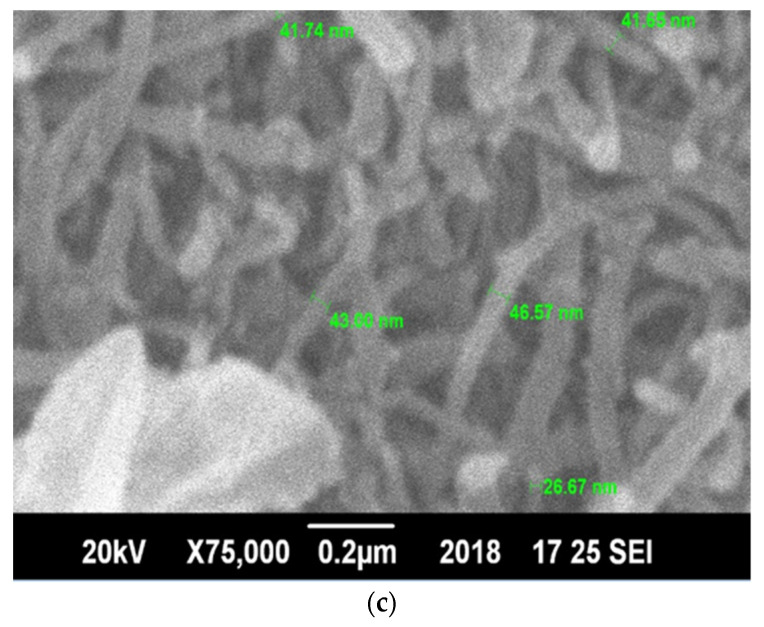
Scanning electron micrographs of synthesized CNTs at different magnifications mentioned on each subfigure (**a**–**c**).

Scanning electron microscopy (SEM) and transmission electron microscopy (TEM) micrographs of synthesized CNTs over the Ni/Mo/Mg (4:0.2:1) catalyst are shown in Figure 12. It was very challenging to ascertain the percent of impurity removed from the SEM image of the CNT, which was purified, as it looks similar to the one of unpurified CNTs. Longer CNTs were produced when the exposure for hydrocarbon was maximized, which was provided by the catalysts at the tip of the CNTs [41]. The size of the synthesized CNTs at ×75,000 magnifications was 26 nm.

**Figure 13:**
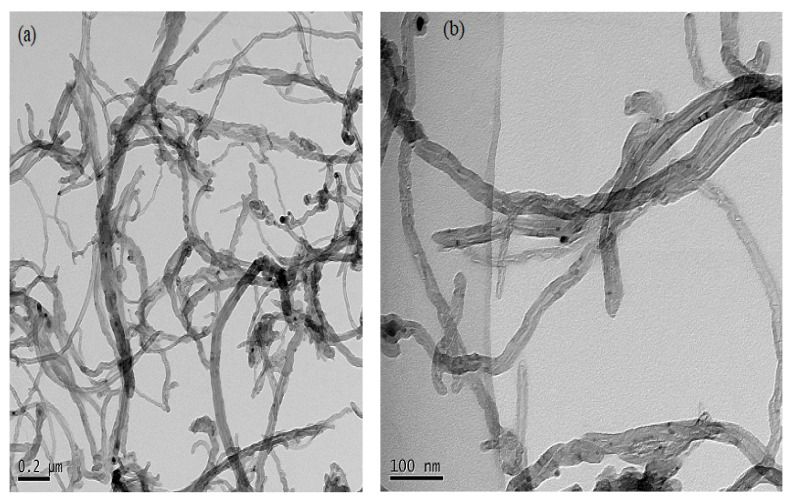

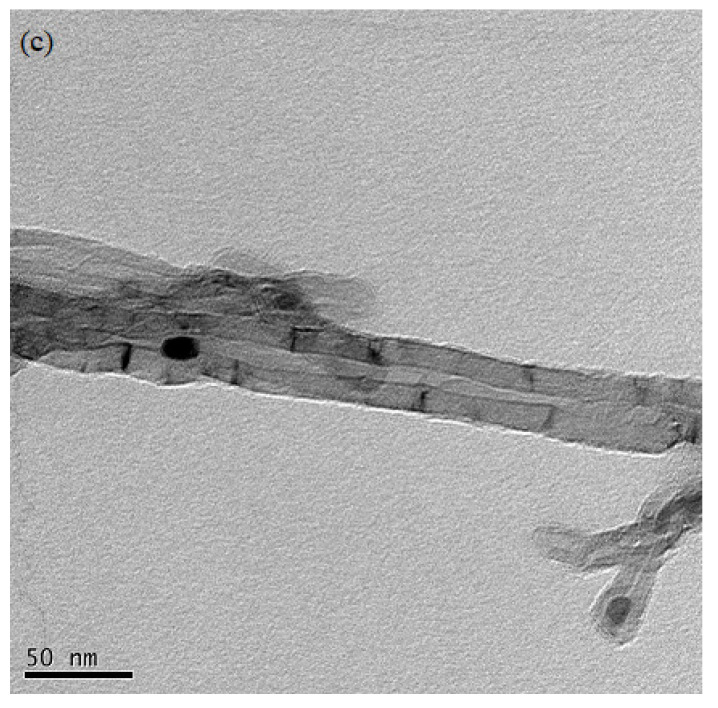
HRTEM images of purified MWCNTs (**a**) 0.2 µm, (**b**) 100 nm, and (**c**) 50 nm.

A high-resolution transmission electron microscope (HRTEM) was used to characterize the size and structure of nanomaterials. Figure 13 shows the CNTs obtained at the growth time of 12 min, using the catalyst Ni4Mo0.2MgO1 which was covered by many graphitized layers. These were (a) 200 nm MWCNTs with well-aligned graphene walls (b) 100 nm with distorted graphene walls and (c) 50 nm MWCNTs. Thus, the purity and graphitization of CNTs were enhanced with growth time. The TEM images showed the purified MWCNTs in which the long intertwined CNTs were not entirely clean but most impurities had been removed. Despite purification with acid treatment, the produced CNTs contained a minute amount of impurities such as amorphous carbon, fullerenes, and catalyst particles which could be observed as a black spot in the TEM image as shown in Figure 13. Thus, the purification for MWCNTs was not very effective in this study, which is a serious issue if CNTs need to be used directly as a functional filler in composite materials. TEM observations show that the most probable diameters of nanotubes are around about 50 nm.

### 3.8. Adsorption of Cr (VI)

Synthesized MWCNTs were used to remove chromium from synthetic wastewater. Using MWCNTs in 2.8 h, pH = 3, and rpm = 400, approximately 81.83% of Cr (VI) was removed for a quantity of 2–8 mg of CNTs. Using MWCNTs, the amount of Cr (VI) removed from wastewater was approximately 78.8% [42]. To remove heavy metals from aqueous solution, as per these studies, the CNTs are excellent adsorbents.

#### 3.8.1. Effect of pH

**Figure 14:**
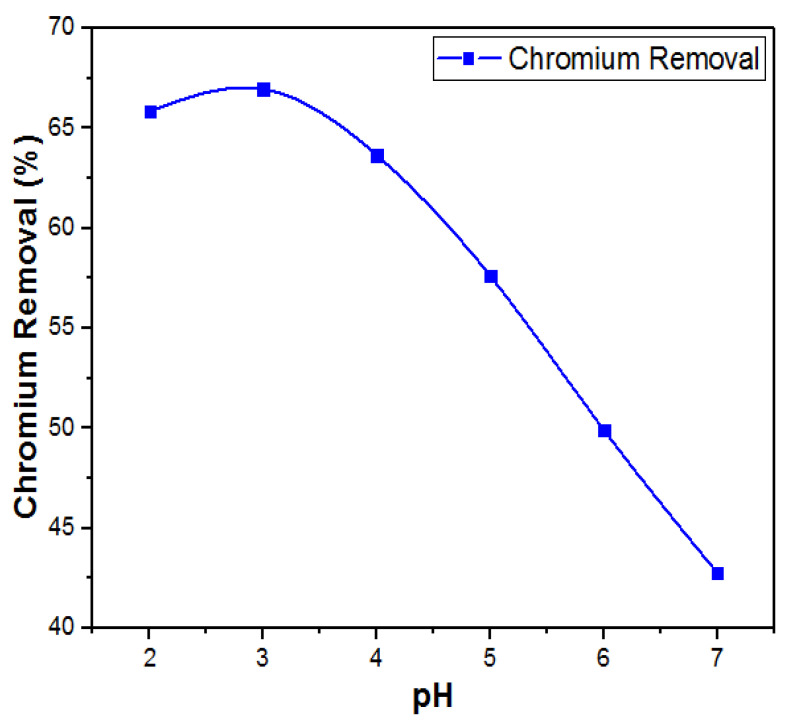
Effects of pH on removal of Cr (VI).

For controlling the Cr (VI) adsorption process, the system pH was observed as one of the key parameters in this study. The maximum removal efficiency of Cr (VI) was higher at a low pH as shown in Figure 14. The optimum pH was observed at pH 3.0 and the remaining trials were performed at this pH. With an increase in pH, the negative charge on the CNTs’ surface increased. Due to this, repulsion was observed between the CNTs and Cr (VI). Therefore, with an increase in pH a decrease in removal efficiency was observed. [43]. Different results were obtained with varying pH values. This means the removal process was highly related to the variations in pH of the solution. It was observed that as the pH values increased from 2.0 to 7.0 the adsorption capacity decreased. This observable fact is explained due to the presence of different forms of Cr (VI) in the aqueous phase. The dominant forms of Cr (VI) were Cr_2_O_7_^−2^ and HCrO_4_^−2^ ions in the pH range of 2–7. MWCNTs’ surfaces, at low pH, became positively charged due to the protonation effect and thus Cr (VI) adsorption was enhanced due to the electrostatic forces between the MWCNTs and the negatively charged Cr_2_O_7_^−2^ and HCrO_4_^−2^ ions [44]. While CrO_4_^−2^ ions prevailed in the solution at higher pH values, the MWCNTs surface protonation decreased, and the adsorption efficiency was thus decreased [45].

#### 3.8.2. Effect of Adsorbent Dosage on Chromium Removal

**Figure 15:**
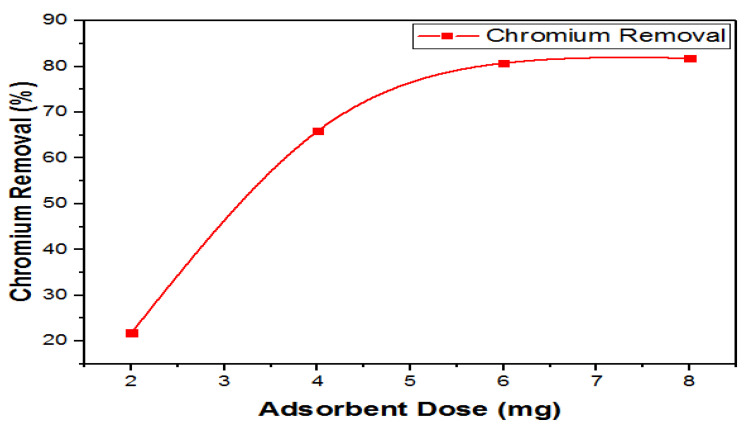
Effects of adsorbent dosage on removal of Cr (VI).

With an increase in the adsorbent dosage from 2 to 8 mg, keeping all the other parameters constant, the removal of Cr (VI) increased. This is illustrated in Figure 15. The highest removal rate was observed at low concentrations of the adsorbent. This is because the number of active sites is higher at lower adsorbent concentrations. However, it decreased as the aggregation of particles occurred at a higher concentration of the adsorbent dosage.

In the higher adsorbent dosage, in contrast to the lower dosage, the capacity of adsorption was not fully utilized. Consequently, it is possible that with an increase in adsorbent dosage the capacity of adsorption may decrease [46].

#### 3.8.3. Effect of Adsorption Contact Time

**Figure 16:**
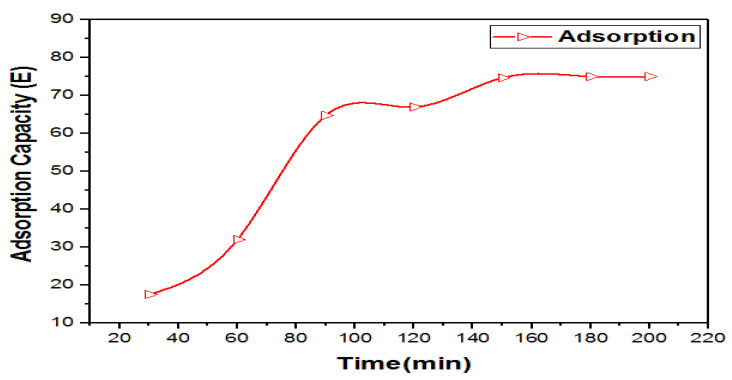
Effect of contact time on the adsorption process.

The removal efficiency of Cr (VI) was increased to 81% as the time of adsorption increased from 30 to 200 min at pH 3 and an adsorbent (MWCNTs) dose of 8 mg. The maximum removal was noted at 180 min of adsorption time as shown in Figure 16. The surface coverage of the adsorbent was higher as time progressed, and no further adsorption occurred [30].

## 4. Conclusions

Poultry litter was used as a hydrocarbon source for CNT production. The Response Surface Methodology (RSM) was adopted to optimize the Ni/Mo/MgO mole ratio. Among them, Ni metal was found to have the highest proportion as compared to Mo and MgO. Poultry waste as a hydrocarbon source was combusted in the presence of a Ni/Mo/MgO catalyst in an electrically heated tube furnace for the growth of CNTs. A high yield of CNTs was obtained using an optimized molar ratio (Ni/Mo/MgO (4:0.2:1) of catalytic precursors at 825 °C, 4 g poultry litter weight, 100 mg catalyst weight, with 12 min of combustion time.

The synthesized CNTs were used in wastewater treatment for the removal of Cr (VI) because MWCNTs exhibited excellent adsorption properties. The adsorption efficiency by MWCNTs was increased with a high adsorbent dosage, but the equilibrium adsorption capacity decreased considerably. As the pH value was increased the adsorption capacity was found to decrease. A maximum adsorption of Cr (VI) from synthetic wastewater by synthesized MWCNTs was noted at 82.0%. Adsorbed chromium can be used as a catalyst. Chromium-based catalysts are highly valuable for ethylene polymerization, and oligomerization catalysts are broadly applied for the industrial production of polyethylene and 1-hexene.

## Figures and Tables

**Table 1:** Moisture content of poultry litter.

Sr. No	Weight of PL before Drying (W2) (gm)	Weight of PL after Drying (W1) (gm)	Moisture Content (%)
1.	200	89.64	55.18
2.	200	82.28	58.88
3.	200	103.7	48.5
		Mean = 91.87 S.D = 10.88	Mean = 54.18 S.D = 5.26

**Table 2:** Elemental analysis of poultry litter.

Elements	C	N	O	Na	Mg	Si	S	Cl	K	Ca
Conc.	65.17	1.55	18.77	0.70	0.50	0.04	0.08	0.72	1.71	0.98

**Table 3:** Optimization of Ni/Mo/MgO concentrations in the catalyst.

Run	A: Nickel (Mol)	B: Molybdenum (Mol)	C: Magnesium Oxide (Mol)	Carbon Yield (%)
1.	2.00	0.20	2.00	24
2.	2.00	0.40	2.00	21
3.	2.00	0.60	2.00	24
4.	2.00	0.40	1.00	25
5.	4.00	0.40	2.00	39
6.	4.00	0.60	1.00	40
7.	4.00	0.20	3.00	43
8.	4.00	0.40	2.00	38
9.	4.00	0.40	2.00	38
10.	4.00	0.60	3.00	35
11.	4.00	0.20	1.00	44
12.	6.00	0.20	2.00	37
13.	6.00	0.60	2.00	32
14.	6.00	0.60	3.00	32
15.	6.00	0.40	1.00	39

**Table 4:** ANOVA for Box–Behnken design for change in carbon yield due to different mole ratios of the catalyst precursors.

Source	Sum of Squares	df	Mean Square	F-Value	*p*-Value	Significance
**Model**	763.34	9	84.82	117.97	<0.0001	significant
A-Ni	277.32	1	277.32	385.72	<0.0001	–
B-Mo	49.16	1	49.16	68.38	0.0004	–
C-Mg	11.93	1	11.93	16.59	0.0096	–
AB	0.6131	1	0.6131	0.8528	0.3981	–
AC	1.08	1	1.08	1.51	0.2743	–
BC	3.30	1	3.30	4.59	0.0851	–
A²	335.36	1	335.36	466.45	<0.0001	–
B²	0.0000	1	0.0000	0.0000	0.9959	–
C²	18.96	1	18.96	26.37	0.0037	–
**Residual**	3.59	5	0.7189	–	–	–
Lack of Fit	2.93	3	0.9760	2.93	0.2649	not significant
Pure Error	0.6667	2	0.3333	–	–	–
**Cor Total**	766.93	14	–	–	–	–

R^2^ = 0.9953; Adjusted R^2^ = 0.9869; Predicted R^2^ = 0.9287.

**Table 5:** Process Parameters optimization for CNT growth.

Run	A: Temperature (°C)	B: Time (min)	C: Catalyst Weight (mg)	D: Carbon Yield (%)
1.	700.00	12.50	80.00	13.24
2.	700.00	12.50	120.00	13.85
3.	700.00	5.00	100.00	14.12
4.	700.00	20.00	100.00	15.51
5.	825.00	20.00	80.00	30.37
6.	825.00	5.00	80.00	30.86
7.	825.00	20.00	120.00	31.02
8.	825.00	5.00	120.00	32.1
9.	825.00	12.50	100.00	44.04
10.	825.00	12.50	100.00	44.38
11.	825.00	12.50	100.00	44.41
12.	950.00	20.00	100.00	16.7
13.	950.00	12.50	80.00	16.72
14.	950.00	12.50	120.00	17.12
15.	950.00	5.00	100.00	18.32

**Table 6:** ANOVA of Box–Behnken design for the dependent variable (carbon yield) in terms of independent variables (different process parameters).

Scheme	Sum of Squares	Df	Mean Square	F-Value	*p*-Value	Significance
**Model**	1975.25	9	219.47	1722.93	<0.0001	significant
A-Temperature	18.42	1	18.42	144.62	<0.0001	–
B-Time	0.4050	1	0.4050	3.18	0.1347	–
C-Catalyst Weight	1.05	1	1.05	8.25	0.0349	–
AB	2.27	1	2.27	17.78	0.0084	–
AC	0.0110	1	0.0110	0.0865	0.7804	–
BC	0.0870	1	0.0870	0.6832	0.4461	–
A²	1784.57	1	1784.57	14,009.47	<0.0001	–
B²	138.73	1	138.73	1089.05	<0.0001	–
C²	184.02	1	184.02	1444.59	<0.0001	–
**Residual**	0.6369	5	0.1274	–	–	–
Lack of Fit	0.5524	3	0.1841	4.36	0.1922	not significant
Pure Error	0.0845	2	0.0422	–	–	–
**Cor Total**	1975.89	14	–	–	–	–

R^2^ = 0.9997; Adjusted R^2^ = 0.9991; Predicted R^2^ = 0.9954; Adeq. Precision = 106.2977.

## Data Availability

The data presented in this study are available on request from the corresponding author.
